# Laser-Based Management of Occupational Photodamage in a Young Adult: A Case Report

**DOI:** 10.7759/cureus.103868

**Published:** 2026-02-18

**Authors:** Luis L Velázquez Arenas, Sarahi Garay Enriquez, Daniela Gómez Guerra

**Affiliations:** 1 Dermatology, School of Medicine and Health Sciences at Tecnológico de Monterrey, Monterrey, MEX; 2 Medicine, School of Medicine and Health Sciences at Tecnológico de Monterrey, Monterrey, MEX

**Keywords:** photo-damaged skin, photorejuvenation, picosecond laser, qs ruby laser, solar lentigines

## Abstract

Cutaneous photodamage results from chronic ultraviolet exposure and represents a frequent concern in dermatologic practice. Laser-based therapies play a central role in its management, particularly when combined in a multimodal, individualized approach. We report the case of a 32-year-old woman with occupational sun exposure treated with a staged laser protocol incorporating a picosecond 1064 nm laser for global photorejuvenation and pigment modulation and a Q-switched ruby laser (694 nm) for solar lentigines. The patient achieved sustained clinical improvement with high satisfaction and no significant adverse events. This case supports the use of personalized, multimodal laser strategies for a well-tolerated and effective management of photodamage and associated pigmentary conditions.

## Introduction

Ultraviolet radiation is classified into three wavelength-dependent categories: ultraviolet C (UVC), with wavelengths between 100 and 290 nm; ultraviolet B (UVB), ranging from 290 to 320 nm; and ultraviolet A (UVA), from 320 to 400 nm. UVC radiation is largely attenuated by the ozone layer, limiting cutaneous exposure. In contrast, both UVB and UVA penetrate the atmosphere and are responsible for cutaneous ultraviolet exposure, with UVB accounting for up to 95% of the ultraviolet radiation reaching the skin [1].

Cutaneous photodamage refers to the spectrum of structural and biological alterations that develop following exposure to ultraviolet radiation. UVB radiation plays a central role in acute skin injury and is strongly associated with skin sunburn, carcinogenesis, inflammatory responses, photo-induced immunosuppression, and stimulation of melanogenesis. On the other hand, UVA radiation contributes primarily to chronic cutaneous changes, including premature skin aging, dryness, hyperpigmentation, and skin sensitization reactions [2].

Laser-based therapies have become a cornerstone in the management of photodamaged skin due to their ability to induce dermal remodeling and selectively target pigmentary abnormalities [3]. Here, we present a case of a 32-year-old woman with chronic occupational sun exposure managed with a personalized, multimodal laser protocol, achieving sustained clinical improvement and high patient satisfaction without significant adverse events.

## Case presentation

**Figure 1:**
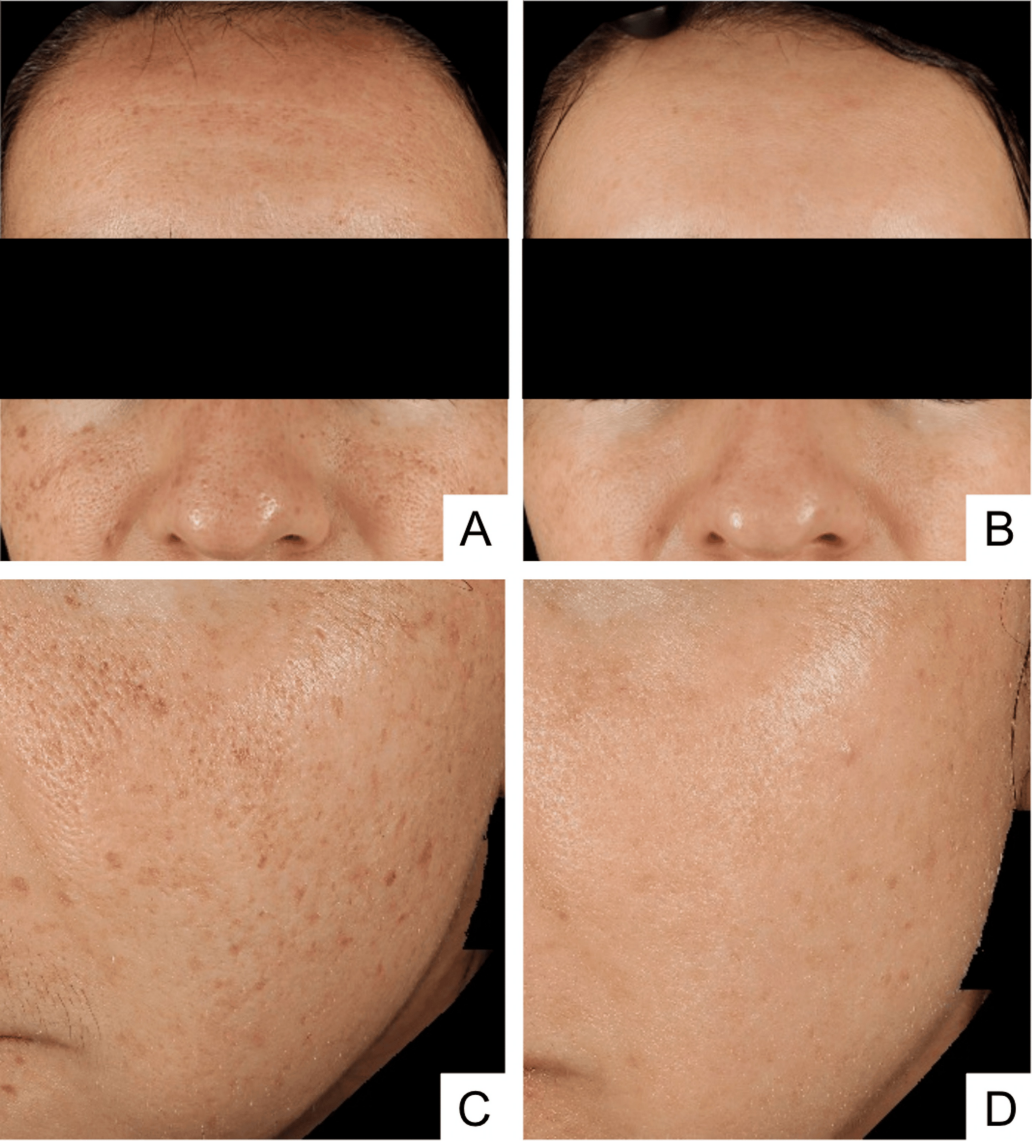
Before and after multimodal laser treatment. Panels A and C were obtained prior to initiation of laser treatment in June 2024. Panels B and D were obtained in January 2026, demonstrating sustained clinical improvement.

A 32-year-old female professional soccer player with a Fitzpatrick skin phototype IV and a history of chronic occupational sun exposure, beginning at three years of age and currently estimated at approximately three hours per day, presented for evaluation and treatment of facial photodamage. Clinical examination revealed fine lines, solar elastosis, textural irregularities, dyspigmentation, telangiectasias, and solar lentigines consistent with cumulative ultraviolet-induced cutaneous changes. Given the patient’s occupational exposure and preference for a moderate intensity, technology-based approach, a staged laser rejuvenation plan was designed. In June 2024, the patient underwent an intermediate facial rejuvenation session using a picosecond 1064 nm laser (Discovery Pico®; Quanta System, Samarate, Italy), with an 8 mm fractional handpiece at 10 Hz and fluence of 0.5 J/cm². Topical anesthetic was applied under occlusion for one hour prior to the procedure. The treatment was well tolerated, and no immediate adverse effects were observed. Post-procedure care included application of Cicaplast®, mineral sunscreen, thermal spring water, gentle cleansing with a neutral soap, and temporary suspension of her regular skincare products for one week. In January 2025, the patient returned for a second laser session focused on rejuvenation of the face and neck. A picosecond 1064 nm laser with an 8 mm fractional handpiece was used at 10 Hz and 0.45 J. By patient preference, treatment of the neck was limited to the upper half. In this session, the patient reported subjective improvement in skin texture and tone, coinciding with a temporary reduction in sun exposure. In October 2025, the patient presented with solar lentigines, for which a pigment-targeted, multimodal laser treatment protocol was initiated. Treatment included picosecond 1064 nm laser toning at 10 Hz and 0.67 J, delivering 7,747 pulses to the face. Facial lentigines were treated with a Q-switched ruby laser at 694 nm, 1 Hz, and 4.6 J. The patient was counseled extensively on strict photoprotection and advised against intentional tanning. One month later, a second pigment-targeted laser session was performed using a similar multimodal approach with adjusted parameters. Picosecond 1064 nm laser toning was used delivering 6,000 pulses to the face, followed by fractional picosecond treatment with a 9 mm handpiece at 0.2 J in a single pass over the entire facial surface. Facial lentigines were retreated with a Q-switched ruby laser at a fluence of 3.6 J. Throughout the treatment course, no significant complications were observed, and the patient reported high satisfaction with both tolerability and outcomes (Figure 1A-1D). 

Objective assessment using the Janus Facial Skin Analysis System was performed to compare baseline and post-treatment parameters, including pores, wrinkles, elasticity, pigmentation, UV pigmentation, and skin tone. Improvement was documented in pore size, elasticity, pigmentation, and UV pigmentation. For pores, the age-matched reference value was 32, decreasing from 33 at baseline to 30 after treatment. Elasticity showed a marked change relative to the reference value of 46, improving from 54 to 38. Pigmentation (reference 20) decreased from 35 to 33, and UV pigmentation (reference 18) decreased from 43 to 41. Overall skin tone remained stable, and although the patient reported a subjective improvement in fine lines, objective analysis demonstrated an increase in the wrinkle score. Given the patient’s ongoing occupational sun exposure, she remains under close dermatologic follow-up at two-to-three-month intervals, with a maintenance plan that includes strict photoprotection and periodic laser-based interventions.

## Discussion

Multiple clinical studies have demonstrated the safety and efficacy of picosecond lasers operating at wavelengths of 532, 755, and 1064 nm in the management of facial photodamage and pigmentary lesions. Although picosecond laser technology was initially developed for the removal of unwanted tattoos, its clinical utility has expanded substantially. Regarding discrete pigmented lesions, these systems have been applied to conditions such as solar lentigines, ephelides, café-au-lait macules, nevus of Ota, Hori’s macules, and verrucous epidermal nevi. With accumulating evidence supporting their role in global photorejuvenation and improvement of overall skin quality, this versatility, together with a favorable safety profile, supports the integration of picosecond lasers into comprehensive, multimodal laser-based rejuvenation strategies [4]. In this context, Tang et al. (2025) conducted a randomized split-face study comparing fractional 1064 nm Nd:YAG picosecond laser and fractional Q-switched 1064 nm Nd:YAG laser for the treatment of facial photoaging. After five treatment sessions, both modalities produced statistically significant improvements in wrinkles, pore size, and skin texture, as objectively quantified by VISIA analysis and supported by reflectance confocal microscopy findings. No significant differences in clinical efficacy were observed between the two laser systems, supporting the role of both fractional picosecond and Q-switched Nd:YAG lasers as effective and safe options [5].

Based on the prospective study by Sadighha et al. (2008), Q-switched ruby laser (694 nm) shows high efficacy in the treatment of solar lentigines, achieving complete clearance in all treated lesions after one or two sessions, regardless of Fitzpatrick skin type II-IV. Although Q-switched ruby laser has a low incidence of adverse effects, its use in darker skin types has been associated with a higher risk of hypopigmentation and post-inflammatory hyperpigmentation (PIH). In this study, PIH occurred in approximately 10% of patients; however, all pigmentary alterations were transient and resolved within six months of follow-up [6]. Additionally, Imhof et al. (2016) conducted a prospective trial comparing the Q-switched ruby laser with a triple combination topical therapy (TCT) containing hydroquinone, tretinoin, and dexamethasone for the treatment of solar lentigines. The study demonstrated that both Q-switched ruby laser and TCT resulted in clinically meaningful pigment lightening; however, Q-switched ruby laser achieved significantly faster and superior clearance. Regarding safety, both treatments were generally well tolerated; however, Q-switched ruby laser was associated with a higher incidence of transient crusting and hyperpigmentation compared with TCT. Nevertheless, all side effects resolved by the final follow-up, supporting the favorable safety profile of Q-switched ruby laser when appropriately selected and monitored [7].

## Conclusions

This case illustrates that a personalized, multimodal laser approach combining picosecond and Q-switched ruby laser technologies can effectively address photodamage and pigmentary lesions in patients with chronic sun exposure. Careful selection of laser modalities, staged treatment planning, and reinforcement of strict photoprotection were associated with favorable tolerability and patient satisfaction in this case. These findings align with existing evidence supporting the safety and efficacy of laser-based therapies in the management of complex photodamage and pigmentary disorders.
